# Radiomics-based prediction of survival in patients with head and neck squamous cell carcinoma based on pre- and post-treatment ^18^F-PET/CT

**DOI:** 10.18632/aging.103508

**Published:** 2020-07-16

**Authors:** Zheran Liu, Yuan Cao, Wei Diao, Yue Cheng, Zhiyun Jia, Xingchen Peng

**Affiliations:** 1 Department of Biotherapy, Cancer Center, West China Hospital of Sichuan University, Chengdu 610041, China; 2 Department of Nuclear Medicine, West China Hospital of Sichuan University, Chengdu 610041, China; 3 Department of Radiology, West China Hospital of Sichuan University, Chengdu 610041, China

**Keywords:** F-PET/CT ^18^, head and neck squamous cell carcinoma, radiomics, prognosis, machine learning

## Abstract

Background: 18-fluorodeoxyglucose positron emission tomography/computed tomography (^18^F-PET/CT) has been widely applied for the imaging of head and neck squamous cell carcinoma (HNSCC). This study examined whether pre- and post-treatment ^18^F-PET/CT features can help predict the survival of HNSCC patients.

Results: Three radiomics features were identified as prognostic factors. Radiomics score calculated from these features significantly predicted overall survival (OS) and disease-free disease (DFS). The clinicopathological characteristics combined with pre- or post-treatment nomograms showed better ROC curves and decision curves than the nomogram based only on clinicopathological characteristics.

Conclusions: Combining clinicopathological characteristics with radiomics features of pre-treatment PET/CT or post-treatment PET/CT assessment of primary tumor sites as positive or negative may substantially improve prediction of OS and DFS of HNSCC patients.

Methods: 171 patients who received pre-treatment ^18^F-PET/CT scans and 154 patients who received post-treatment ^18^F-PET/CT scans with HNSCC in the Cancer Imaging Achieve (TCIA) were included. Nomograms that combined clinicopathological features with either pre-treatment PET/CT radiomics features or post-treatment assessment of primary tumor sites were constructed using data from 154 HNSCC patients. Receiver operating characteristic (ROC) curves and decision curves were used to compare the predictions of these models with those of a model incorporating only clinicopathological features.

## INTRODUCTION

Head and neck cancer, which manifests most often as head and neck squamous cell carcinoma (HNSCC), is the sixth most common malignancy, with an incidence of 650,000 cases and 330,000 deaths annually worldwide [1, 2]. HNSCC refers to a broad range of malignant tumors, including in the oral cavity, larynx, oropharynx, and hypopharynx [3]. The 5-year survival rate of patients with HNSCC is only about 60% and is lowest for those with tumors in the hypopharynx [4]. HNSCC is usually treated by surgical resection with or without adjuvant radiotherapy or by definitive radiotherapy with or without concurrent chemotherapy [5].

The stage of HNSCC, which is vital for guiding treatment decisions, is usually determined based on imaging of the head and neck with computed tomography (CT) or magnetic resonance imaging (MRI) [6]. Increasingly, 18-fluorodeoxyglucose (^18^F-FDG) positron emission tomography/CT, which provides both anatomical and metabolic information, is used to distinguish benign from malignant disease, assess treatment response and detect recurrence [7, 8]. PET/CT can detect HNSCC with a sensitivity of 72-96% and specificity of 83-100% [8–10]. While this imaging modality continues to gain ground as a tool for diagnosing disease and assessing treatment response, whether it can predict patient prognosis is unclear.

It may be possible to predict prognosis based on quantifiable features in PET/CT scans taken before or after treatment [11–13]. For example, studies have linked the survival of patients with HNSCC, lymphoma, or non-small-cell lung cancer to mean and maximum standardized ^18^F-FDG uptake values (SUV*mean*, SUV*max*), metabolic tumor value (MTV) and total lesion glycolysis (TLG). These PET/CT features reflect tumor metabolic activity and lesion size [14–18]. Using radiomics to predict the prognosis of cancer patients is in its infancy, so the consensus still lacks on what image features provide the most reliable predictions.

In the present study, we used a quantitative radiomics approach to extract imaging features from pre-treatment ^18^F-FDG PET/CT scans of patients with HNSCC and a conventional approach to extract positive/negative findings from post-treatment scans. Then we combined each of these types of data with clinicopathological characteristics to generate models to predict survival. The predictive performance of these models was compared to that of a model-based only on clinicopathological characteristics.

## RESULTS

### Patient characteristics and radiomic signatures

**Figure 1:**
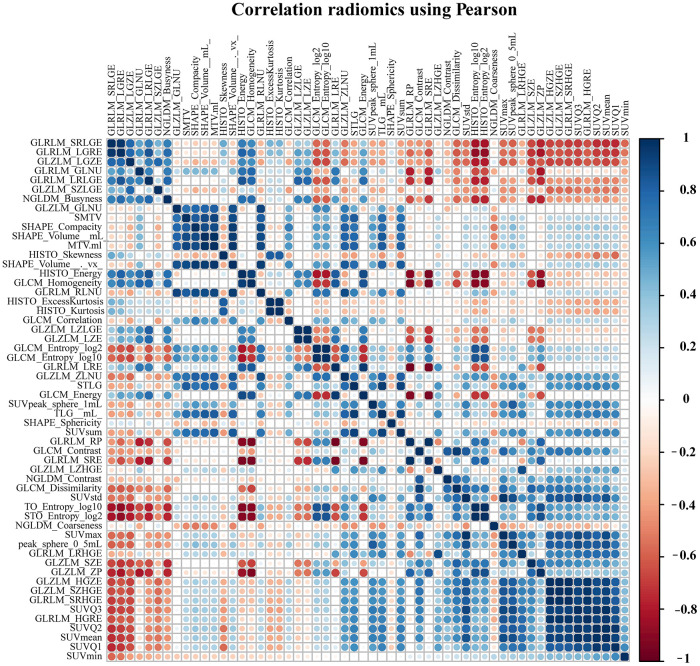
**The correlation matrix between pre-processing radiomics features.**

**Figure 2:**
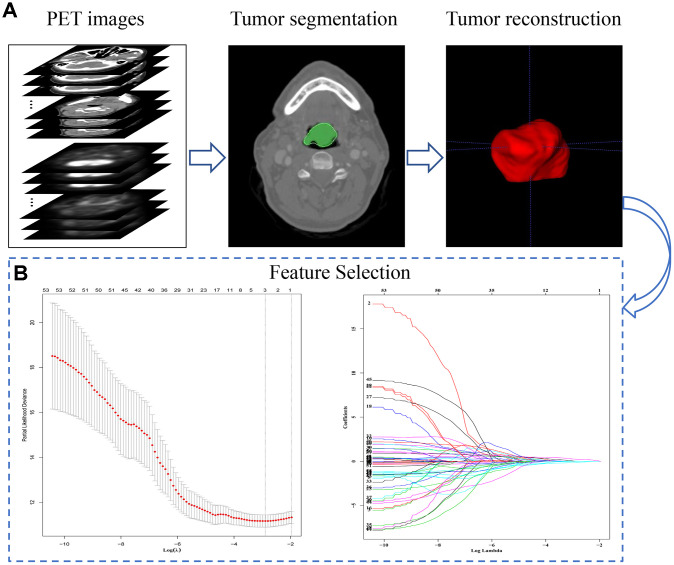
**The extraction process and general characteristics of pre-treatment PET signatures with HNSCC patients.** (**A**) The segmentation and reconstruction process of PET/CT images. (**B**) Demonstration of the varies of Lasso coefficient in different log (λ) sequence. A 15-fold cross validation were used to select the most optimal penalty parameter λ via minimum criteria. The minimum λ (λ = 0.05209914) were chose according to the criteria. Abbreviations: OS: overall survival. DFS: disease free survival.

A total of 171 patients (training cohort = 115 and a validation cohort = 56) were analyzed for the construction of a Radiomics score (Rad-score) model based on pre-treatment PET/CT, and 154 patients were analyzed for the development of nomograms based on pre- or post-treatment PET/CT. The clinical characteristics of patients in the training and validation cohorts were summarized in Table 1. The correlations between extracted radiomics features were calculated and visualized by a correlation matrix (Figure 1). LASSO Cox regression was used to choose potential prognostic predictors from the 56 radiomics features in the training cohort (Figure 2). Three radiomics features were identified, and both the univariate and multivariate analyses of the selected features were performed to show the correlation of these features with patients’ survival (Supplementary Table 1). Besides, the collinearity statistics demonstrated that the collinearity between selected features was acceptable (variance inflation factor: SHAPE_Sphericity: 1.102, NGLDM_Coarseness: 1.274, SMTV: 1.375). Then, these features were used to calculate Rad-score for each patient:

**Table 1:** Demographics and clinicopathologic characteristics of patients with HSNCC.

**Variables**	**Training cohort (N = 115)**		**Validation cohort (N = 56)**	
	**N**	**%**	**N**	**%**
**Gender**				
Male	100	86.96	47	83.93
Female	15	13.04	9	16.07
**Age (years)**				
60	71	61.74	30	53.57
≥ 60	44	38.26	26	46.43
**Tumor size**				
≤ 4	94	81.74	27	48.21
4	57	49.57	29	51.79
**Tumor Location**				
Oropharynx	92	80.00	47	83.93
Larynx	13	11.30	6	10.71
Oral cavity	2	1.74	2	3.57
Hypopharynx	8	6.96	3	5.36
**Differentiation status**				
Well	13	11.30	4	7.14
Moderate	51	44.35	28	50.00
Poor and undifferentiated	51	44.35	24	42.86
T stage *				
T1	21	18.26	10	17.86
T2	37	32.17	17	30.36
T3	34	29.57	18	32.14
T4	23	20.00	11	19.64
N stage *				
N0	10	8.70	9	16.07
N1	11	9.57	8	14.29
N2a	5	4.35	4	7.14
N2b	56	48.70	19	33.93
N2c	28	24.35	9	16.07
N3	5	4.35	7	12.50
TNM stage *				
I	1	0.87	0	0.00
II	2	1.74	3	5.36
III	15	13.04	13	23.21
IVA	90	78.26	32	57.14
IVB	7	6.09	8	14.29

* according to 7^th^ AJCC stage system.

Abbreviation: HSNCC-head and neck squamous cell carcinoma; N-number.

$$\begin{array}{c} Rad–score=–0.3392*SHAPE\_Sphericity+0.3736*\\ NGLDM\_Coarseness+1.5655*SMTV \end{array}$$

The optimal cut-off value of the Radscore was 0.01187901, and patients in the training and validation cohorts were accordingly classified as low- or high-risk. Supplementary Table 2 shows clinicopathological characteristics between patients with low and high risk.

**Figure 3:**
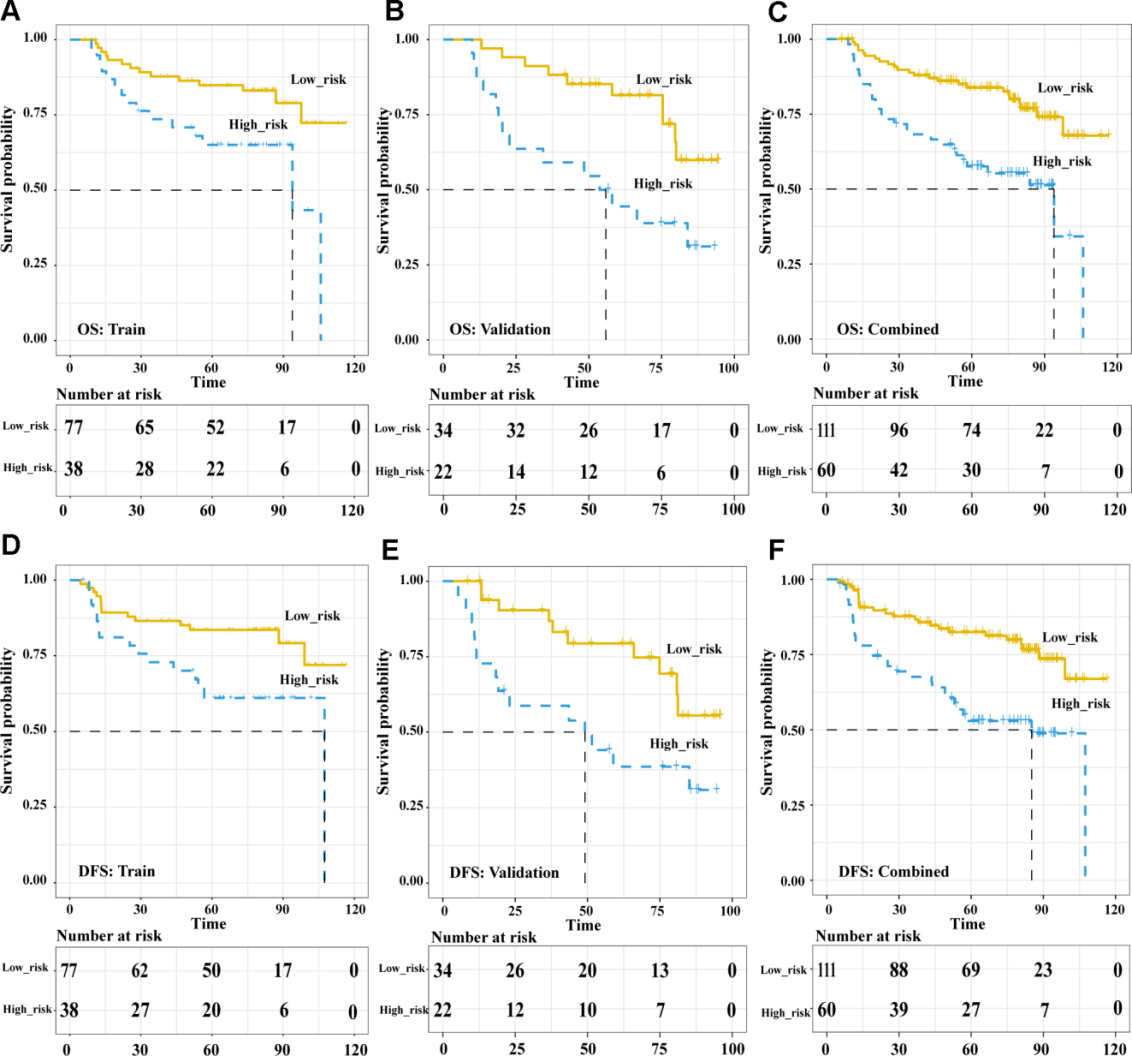
**The Pre-treatment PET signatures could significantly stratify patients’ OS and DFS.** Kaplan-Meier survival analysis of pre-treatment Rad-score-defined risk levels in the training, validation cohorts and combined cohort. OS: the training cohort (**A**), validation cohort (**B**), and combined cohort (**C**). DFS: the training cohort (**D**), validation cohort (**E**), and combined cohort (**F**). Abbreviations: OS: overall survival. DFS: disease free survival.

**Figure 4:**
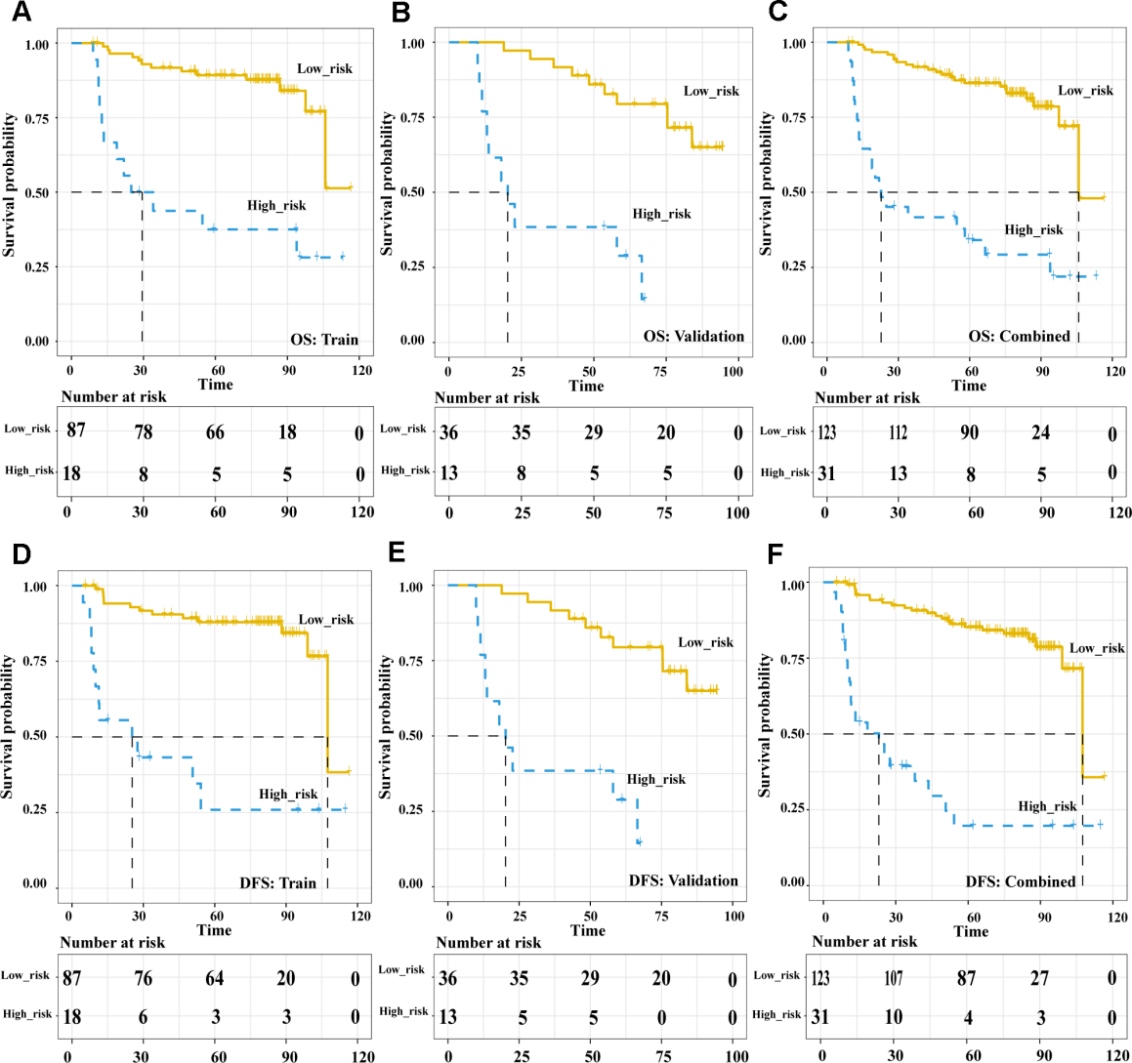
**The Post-treatment PET outcomes is a powerful tool to stratify patients’ OS and DFS.** Kaplan-Meier survival analysis of post-treatment PET-outcome-defined risk levels in the training, validation cohorts and combined cohort. OS: the training cohort (**A**), validation cohort (**B**), and combined cohort (**C**). DFS: the training cohort (**D**), validation cohort (**E**), and combined cohort (**F**). Abbreviations: OS: overall survival. DFS: disease free survival.

In the pre-treatment Rad-score model, the Kaplan-Meier analysis showed that high risk was associated with significantly worse overall survival (OS) in the training cohort (HR 5.89, 95%CI 1.74-20.02, *p* = 0.004), validation cohort (HR 5.59, 95%CI 1.83-17.09, *p* = 0.003) and both cohorts together (HR 6.33, 95%CI 2.77-14.5, *p* < 0.001). Similar results were obtained for disease-free survival (DFS) in the training cohort (HR 7.04, 95%CI 1.93-25.68, *p* = 0.003), validation cohort (HR 5.10, 95%CI 1.61-16.17, *p* = 0.006) and both cohorts together (HR 6.844, 95%CI 2.90-16.13, *p* < 0.001) (Figure 3). In the post-treatment negative/positive model, Kaplan-Meier analysis showed that a positive finding was significantly related to worse OS (HR 6.609, 95%CI 3.649-11.97, *p* < 0.001) and DFS (HR 8.169, 95%CI 4.453-14.99, *p* < 0.001) (Figure 4). Cox regression showed that both the pre-treatment Rad-score and post-treatment outcomes were significant independent predictors of both OS and DFS (Supplementary Table 3). Besides, we compared the concordance index (C-index, which is proportional to the survival-prediction ability of variables) between Rad-score and four conventional PET features (TLG, MTV, SUV*mean*, and SUV*max*). The results showed that the survival-prediction ability of the Rad-score was much better than not only each single conventional PET feature but also the combined of four (Supplementary Table 4).

### Prediction of OS and DFS using models based on radiomic signatures

As a first step in constructing predictive models based on radiomic signatures, we created a conventional prediction model based only on clinical characteristics of 154 HNSCC patients according to inclusion and exclusion criteria. This conventional clinical model also served as a benchmark for assessing the prognostic performance of the radiomic models. The clinical model was constructed by initially including eight clinical characteristics (body mass index, age, T stage, N stage, AJCC stage, cancer site, histology grade, and smoking history), from which age and histology grade were subsequently excluded because they did not satisfy the model’s assumption of proportional hazards. The final clinical model contained body mass index, tumor location, and N stage, while other characteristics were excluded using a stepwise algorithm. This model was used to generate the corresponding reference OS and DFS nomograms (Supplementary Figure 1).

**Figure 5:**
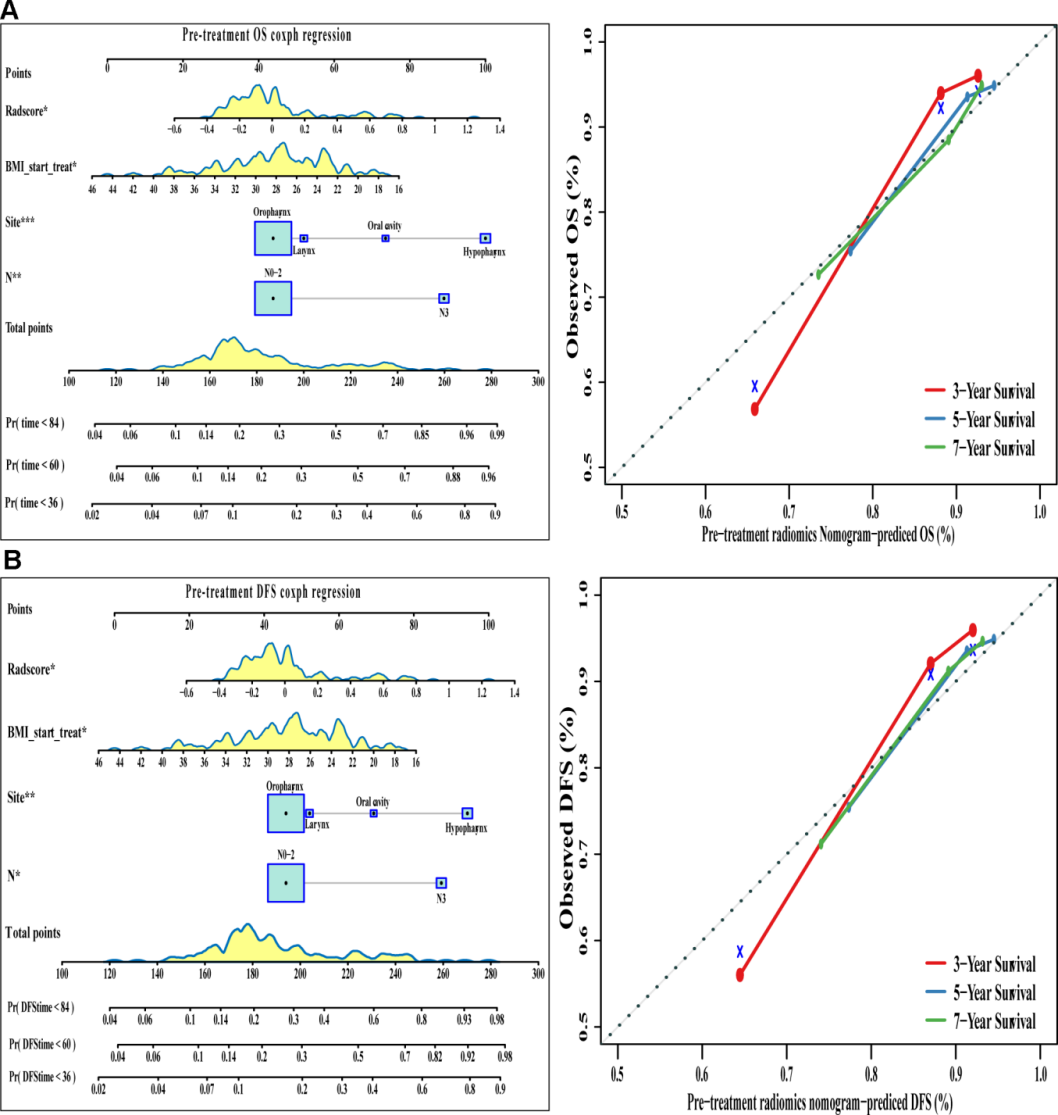
**The visualization of OS and DFS survival models based on pre-treatment Rad-score combined with clinicopathologic characteristics.** The constructed nomograms and their calibration plots to estimate the OS (**A**) and DFS (**B**) in 3, 5, and 7 years. Abbreviations: OS: overall survival. DFS: disease free survival.

Radiomics signatures from the pre-treatment PET/CT scans were added to this conventional clinical model, and the corresponding model was used to generate OS and DFS nomograms (Table 2 and Figure 5). The C index indicated good discrimination of OS (C index 0.77, 95%CI 0.70-0.84) and DFS (C index 0.77, 95%CI 0.70-0.83). Calibration curves calculated for 3, 5, or 7 years showed good agreement with the OS and DFS nomograms.

**Table 2:** Multivariate Cox regression analyses for OS and DFS in the pre-treatment radiomics model and post-treatment PET model.

**Variables**	**Overall survival**		**Disease-free survival**	
	**HR (95%CI)**	***p***	**HR (95%CI)**	***p***
***Pre-treatment Radiomics Model***				
**Rad score**	3.28 (1.23-8.70)	0.017	3.43 (1.24-9.45)	0.017
**N stage (vs. N0-2)**	3.47 (1.40-8.61)	0.007	3.21 (1.29-7.95)	0.012
**Cancer site (vs. Oropharynx)**				
Hypopharynx	4.70 (1.96-11/28)	<0.001	3.90 (1.60-9.48)	0.002
Oral cavity	2.27 (0.30-17.00)	0.425	1.93 (0.26-14.41)	0.521
Larynx	1.25 (0.30-5.14)	0.756	1.19 (0.28-5.12)	0.812
**Start-treatment BMI**	0.93 (0.87-0.99)	0.025	0.924 (0.86-0.99)	0.019
***Post-treatment PET Model***				
**PET outcome (vs. negative)**	6.79 (3.69-12.47)	<0.001	8.26 (4.41-15.44)	<0.001
**N stage (vs. N0-2)**	5.87 (2.67-14.57)	<0.001	5.43 (2.20-13.37)	<0.001
**Cancer site (vs. Oropharynx)**				
Hypopharynx	6.40 (2.60-15.82)	<0.001	5.05 (2.02-12.64)	<0.001
Oral cavity	2.15 (0.28-16.30)	0.461	1.48 (0.20-11.21)	0.700
Larynx	2.17 (0.63-7.55)	0.221	2.33 (0.68-7.99)	0.179
**Start-treatment BMI**	0.91 (0.85-0.98)	<0.001	0.91 (0.85-0.98)	<0.001

**Abbreviations:** OS-overall survival; DFS-disease-free survival; HR-hazard ratio; BMI-body mass index;

PET- positron emission tomography/computed tomography.

**Figure 6:**
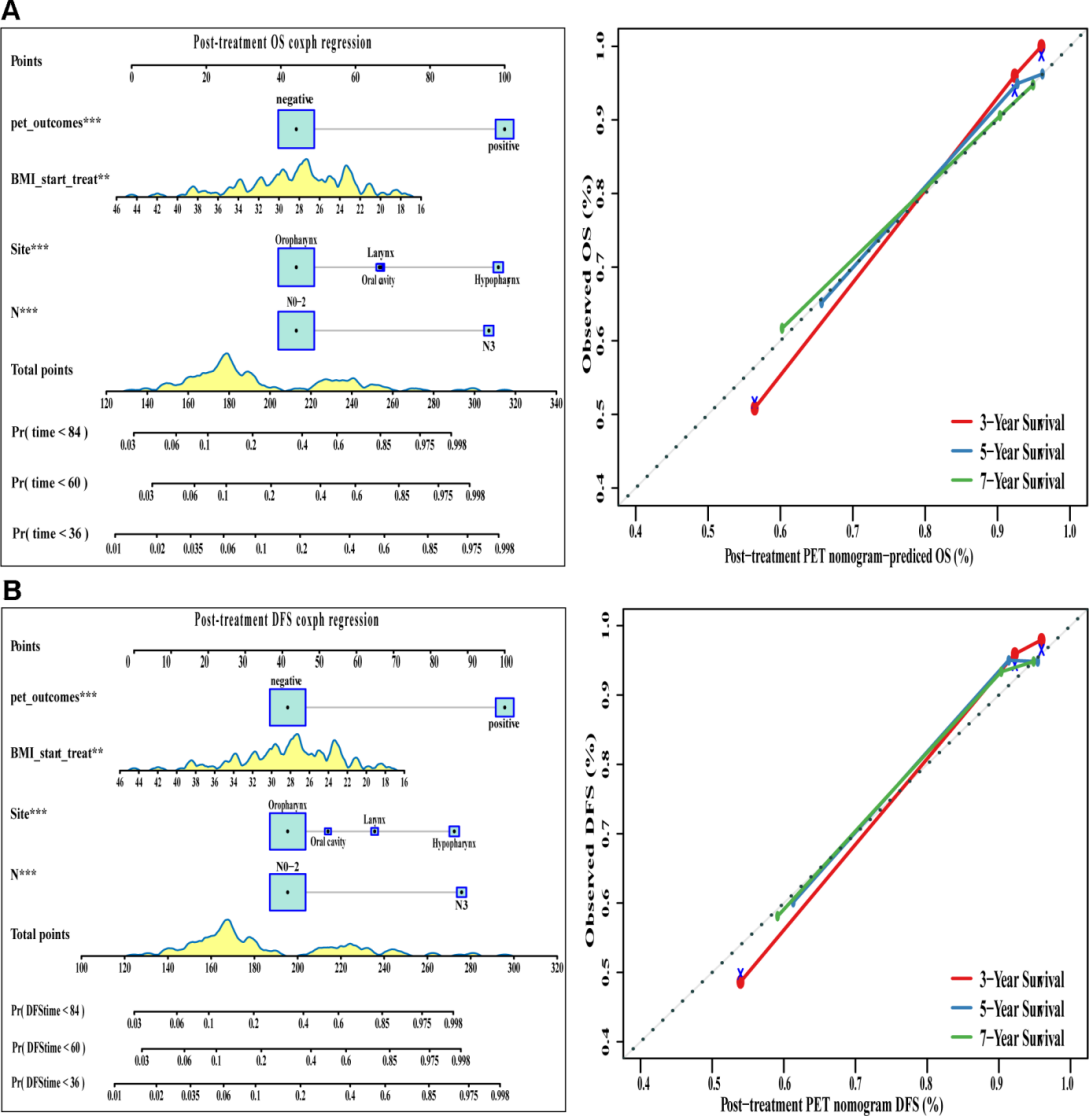
**The visualization of OS and DFS survival models based on post-treatment PET signatures combined with clinicopathologic characteristics.** The constructed nomograms and their calibration plots to estimate the OS (**A**) and DFS (**B**) in 3, 5, and 7 years. Abbreviations**:** OS: overall survival. DFS: disease free survival.

Good results were also obtained when positive/negative findings based on post-treatment PET/CT were added to the conventional clinical model (Table 2 and Figure 6). The corresponding nomograms showed excellent accuracy and discrimination for OS (C index 0.822, 95%CI 0.767-0.877) and DFS (C index 0.832, 95%CI 0.781-0.883).

### Comparison of models

**Figure 7:**
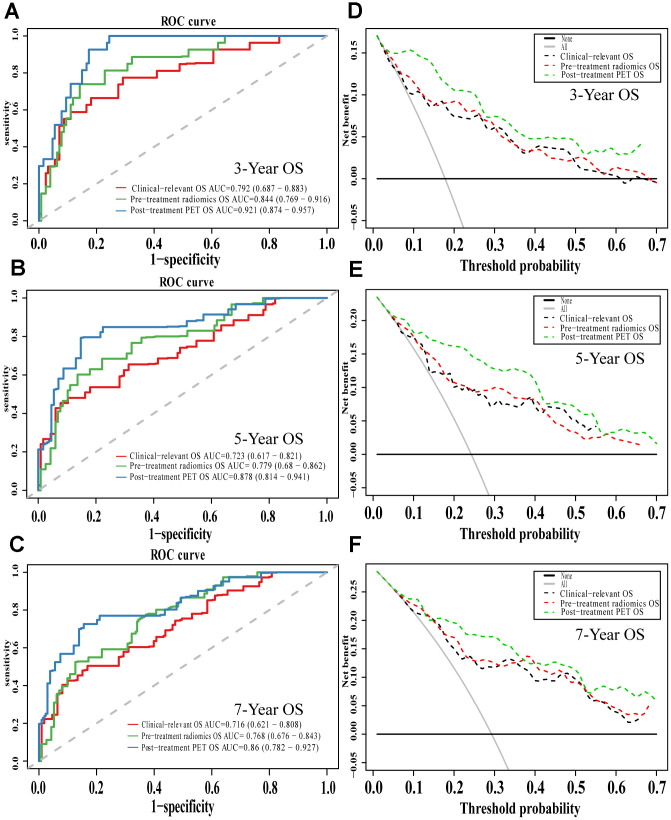
**The evaluation of built OS nomograms.** The ROC curves and DCA curves of the comparison between clinical-relevant, pre-treatment and post-treatment survival OS models in 3, 5, and 7 years. The ROC curves of 3-year survival (**A**), 5-year survival (**B**) and 7-year survival (**C**). The DCA curves of 3-year survival (**D**), 5-year survival (**E**) and 7-year survival (**F**). Abbreviations: OS: overall survival. ROC: receiver operator curve. DCA: decision curve analysis.

**Figure 8:**
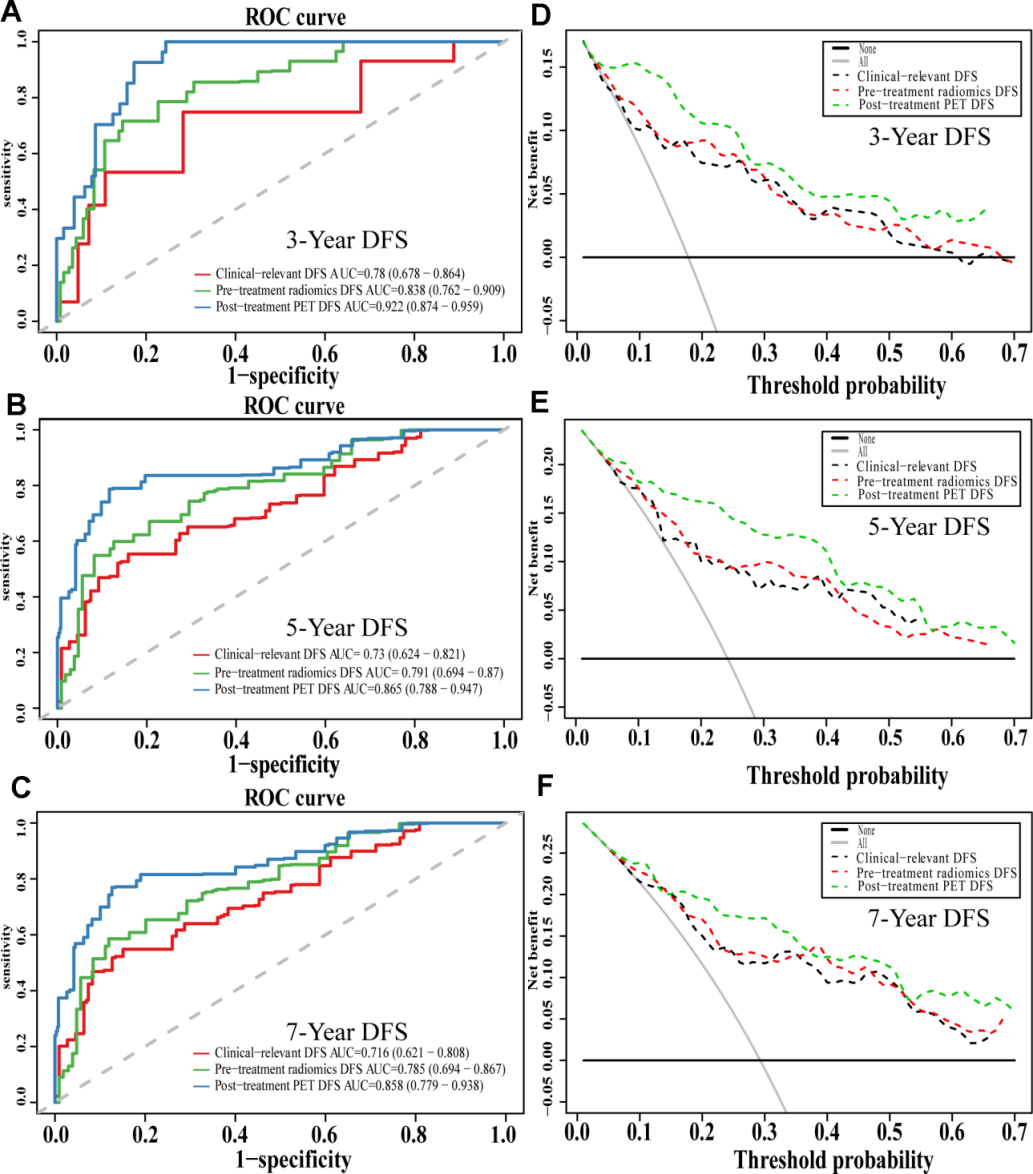
**The evaluation of built DFS nomograms.** The ROC curves and DCA curves of the comparison between clinical-relevant, pre-treatment and post-treatment survival DFS models in 3, 5, and 7 years. The ROC curves of 3-year survival (**A**), 5-year survival (**B**) and 7-year survival (**C**). The DCA curves of 3-year survival (**D**), 5-year survival (**E**) and 7-year survival (**F**). Abbreviations: DFS: disease free survival. ROC: receiver operator curve. DCA: decision curve analysis.

Comparison of ROC curves at 3, 5, and 7 years showed that the pre-treatment model predicted OS and DFS better than the conventional clinical model. In contrast, the post-treatment model performed significantly better than the pre-treatment model. Similarly, decision curves showed that the post-treatment model maximized clinical benefits for patients in the prediction of OS and DFS at 3, 5, and 7 years (Figures 7 and 8).

## DISCUSSION

^18^F-FDG-PET/CT radiomics signatures, which can capture spatial heterogeneity in tumors, have been applied as potential prognostic markers in many cancers, including gastric cancer [19], nasopharyngeal carcinoma [20], NSCLC [21], and HNSCC. HNSCC is a clinically heterogeneous disease, and few biomarkers are available for predicting tumor response to treatment or prognosis [22]. The present study used machine learning to identify 56 radiomics features in PET/CT scans of patients with HNSCC, and these features were significantly associated with OS and DFS. Combining some of these features with patients’ clinicopathological characteristics allowed reliable and accurate predictions of OS and DFS, which were substantially better than those obtained based on clinicopathological characteristics alone. The models described here may help improve the design of treatment strategies in HNSCC and thereby lead to better patient prognosis.

Accurately predicting prognosis is of great importance for optimizing treatment strategies in HNSCC, but it remains controversial. Several studies have attempted to assess the predictive value of radiomics information from CT and MRI images in HNSCC. Koun et al. [23] recruited 62 patients with HNSCC to evaluate the ability of pretreatment CT texture to predict treatment failure in patients with primary HNSCC treated with chemoradiotherapy. They found that three histogram features and four grey-level run length (GLRL) features predicted treatment failure in these patients. Yuan et al. [24] extracted 485 MRI-based radiomic features from 170 patients with HNSCC (85 in the training cohort, 85 in the validation cohort) and obtained higher C indices for the radiomics signature (0.73 for training and 0.71 for validation) and the nomogram (0.76 for training and 0.72 for validation) than the AJCC staging system (0.63 for training and 0.61 for validation). Their study established the feasibility of combining MRI-based radiomic signatures with clinical characteristics to predict prognosis in patients with HNSCC.

^18^F-FDG-PET/CT has also been widely applied to predict survival in cancer patients because of its ability to provide information on tumor burden and aggressiveness. Bogowicz et al. [22] compared PET and CT radiomics for prediction of local tumor control in HNSCC, and they found PET to be more accurate than CT in predicting tumor local control rate. Those authors highlighted the need to pay more attention to PET-based radiomic analysis for predicting prognosis. Kim et al. [25] examined the ability of PET/CT to predict treatment failure and guide clinical decision-making about salvage surgery. Despite their relatively small sample, they were able to predict OS and PFS reasonably well based on post-treatment PET findings. The optimal time to perform PET/CT on HNSCC patients and the optimal prognostic model for predicting survival remain unclear.

Our study identified a pre-treatment Rad-score, comprising SHAPE_Sphericity, NGLDM_Coarseness, and standardized MTV (SMTV). This integrated PET/CT signature, when combined with clinicopathological characteristics, shows promise for predicting OS and DFS of HNSCC patients. Previous studies have demonstrated the prognostic significance of traditional PET quantitative parameters such as SUV [26], MTV, and TLG [27]. We found, however, that these parameters did not predict OS or DFS as well as the combination of our PET/CT radiomic signatures with a subset of clinicopathological characteristics. These findings highlighted the potential role of PET/CT radiomic signatures that could play in the high throughput machine learning era. At the same time, our study suggested that post-treatment positive/negative findings may have even more prognostic potential than pre-treatment Rad-score when combined with clinicopathological characteristics.

Our findings suggested the potential of PET/CT radiomic signatures to predict the prognosis of patients with HNSCC reliably. These promising results may partly reflect our efforts to control for heterogeneity in the patient population, which came from a single center with the same scanner. While this approach allows us to reduce potential confounding due to heterogeneity of patient characteristics and hospital practices, it also threatens the external validity of our results. Therefore, our findings should be verified and extended in larger, preferably multi-site patient populations.

## CONCLUSIONS

The present study using publicly available ^18^F-PET/CT images suggests that combining clinicopathological characteristics with specific radiomic signatures from pre-treatment scans or with post-treatment assessment of primary tumor sites as positive or negative can predict OS and DFS of patients with HNSCC significantly better than clinicopathological characteristics alone.

## MATERIALS AND METHODS

### Patient population

We extracted ^18^F-FDG-PET/CT scans from the publicly available HNSCC dataset on The Cancer Imaging Achieve (TCIA) platform of the University of Texas MD Anderson Cancer Center [28] (http://www.cancerimagingarchive.net/). Of the total set of 2,840 consecutive patients with HNSCC treated with curative radiotherapy at the MD Anderson Cancer Center between 1 October 2003 and 31 August 2013 [29]. Two hundred fifteen patients overlapping in TCGA and TCIA databases were initially selected. Of these, 203 patients were included because they did not have a primary diagnosis of nasopharyngeal carcinoma, cancer of unknown primary site, or recurrent HNSCC. For the identification of pre-radiomics signatures, patients were excluded from the analysis if their pre-treatment PET/CT images were unavailable or the region of interest on their scans was too small to extract features. The rest of the patients were randomly divided into a training cohort and a validation cohort using the *caret* package in R 3.6.1 [30]. Finally, 171 patients with available pre-treatment PET scans and 154 patients with available post-treatment PET/CT scans were included in our study, according to the Data Descriptor [28]. For further identification of post-radiomics signatures and model construction, patients from the original cohort were included except for those who lacked the paired pre- and post-treatment PET/CT images (Supplementary Figure 2).

### Pre-treatment PET/CT image analysis and feature extraction

The pre-treatment PET/CT images were segmented, and the features were extracted using LIFEx 4.0 (http://www.lifexsoft.org) [31]. The primary tumor without lymph nodes was segmented by two specialists in nuclear medicine (Y.C. and W.D.), who delineated a computer-generated volume of interest around voxels equal to or greater than 40% of SUV_max_ [32]. Noise in images was reduced by resampling FDG uptake values using 64 discrete values, boundary SUV values of 0 to 30, and a bin width of 0.47, based on typical SUVs for HNSCC tumors [33]. Data were extracted on 56 quantitative PET parameters, first-order intensity features, shape features, and texture indices (Supplementary Table 5 and Supplementary Material). Finally, texture features were investigated based on gray-level co-occurrence matrices, gray-level run-length matrices, neighborhood gray-tone difference matrix wavelet decompositions, and gray-level size zone matrices.

### Post-treatment PET/CT image interpretation

The post-treatment PET/CT scans were reviewed independently by two specialists in nuclear medicine (Y.C. and W.D.), who determined whether the residual or recurrent disease was presented. Scans were judged negative if no focal increase in FDG uptake was evident, or if an increase in FDG uptake was apparent but could be attributed to physiological causes or the treatment [5]. Discrepancies between the independent assessments were resolved in consultation with a senior specialist in nuclear medicine (Z.Y.J.) and a radiation oncologist (X.C.P.). Pearson correlation analysis was performed to show the correlations between extracted radiomics features.

### Feature selection and integration into a single Rad-score

Post-normalized Fifty-six radiomics features were entered into a “least absolute shrinkage and selection operator” (LASSO) algorithm [34] in a Cox regression model based on penalized maximum likelihood, to shrink the regression coefficients of most radiomics variables to zero. The λ is a penalty parameter that varies in each step of model fitting. Bootstrapping was used to cross-validate 1000 times to the built model and to select the variables most relevant to overall survival (OS) in the training cohort at an optimal λ. The minimum λ giving a minimum mean cross-validated error of the built model was determined, and the coefficients of the selected variables were identified at this λmin. Then a Rad-score for each patient was computed based on all LASSO-selected features using the following formula:

$$\begin{array}{c} Rad–score=\sum_{i=1}^{n}Coefficientoffeature(i)\\ {}*valueoffeature(i) \end{array}$$ (1)

where the coefficient of radiomics feature *(i)* was the coefficient determined in the regression model.

Data in the training set were used to generate a time-dependent receiver operating characteristic (ROC) curve by survivalROC Package in R to describe the ability of the Rad-score to predict OS, which was defined as the period from the first diagnosis to death. This curve was used to identify the optimal cut-off for the Rad-score, and patients whose Rad-scores were higher than this threshold were classified as “high risk,” while those with Rad-scores equal to or lower than the threshold were classified as “low risk”.

### Model construction and evaluation

The following three models were used to predict OS and disease-free survival (DFS), defined as the period from the first diagnosis to death due to HNSCC: a conventional clinical model, a pre-treatment PET/CT model, and a post-treatment PET/CT model. The *conventional clinical model* contained several pre-treatment clinical characteristics that have been linked to the survival of HNSCC patients [35]: body mass index, age, T stage, N stage, stage according to the 7^th^ edition of the American Joint Committee on Cancer (AJCC) guidelines, tumor location, histology grade, and smoking history. The model was optimized in a stepwise manner based on the Akaike information criterion, after which time-dependent variables were excluded by applying an assumption of proportional hazards. The *pre-treatment model* was generated by adding the Rad-score to this conventional clinical model. The *post-treatment model* was created by adding positive/negative findings (based on post-treatment PET/CT scans) to the conventional clinical model. Three nomograms were constructed based on the three models.

The various models were assessed for their ability to predict OS or DFS at 3, 5- or 7-years using calibration curves and Harrell’s concordance index (C index). The “bootstrap split” method [36] was applied with 1000 iterations. Models were also assessed and compared using ROC curves, and the overfitting risk was evaluated using the Akaike information criterion. A decision curve analysis (DCA) was conducted to help to determine which model is the best in clinical use by comparing benefits and the harms of false-positive and false-negative prediction on the same scale [37, 38].

### Statistical analysis

Data were analyzed statistically using R 3.6.1 [30] and a significance threshold of p = 0.05. LASSO-based Cox regression was conducted using the *glmnet* package, while ROC curves and optimal cut-offs were generated using the *survivalROC* and *tdROC* packages [39, 40]. The Pearson’s correlation analysis were conducted and visuliazed by *rattle* package. OS and DFS were calculated, and survival curves were plotted using Kaplan-Meier analysis; statistical inference about the survival difference between high- and low-risk patients was accomplished using the Cox regression statistic, and the analyses were performed using the *survival* package [41, 42]. Multivariate Cox models were constructed and evaluated using the *survival* and *pec* packages [43–45], while decision curves were analyzed using the *DCA* package. When appropriate, results were reported as hazard ratios (HRs) with associated 95% confidence intervals (CIs). Collinearity diagnostics were run using SPSS software, version 25.0 (IBM Corporation, Armonk, NY, USA) to ensure partial regression coefficients derived from regression analyses were estimated precisely and that the relative importance of each predictor for OS and DFS could be assessed reliably.

## Supplementary Material

Supplementary Material

Supplementary Figures

Supplementary Tables
