# Supplementary material for: Radiomics-based prediction of survival in patients with head and neck squamous cell carcinoma based on pre- and post-treatment 18F-PET/CT
Source: Aging (Albany NY). 2020 Jul 16;12(14):14593–619. doi: 10.18632/aging.103508 (PMC7425452; doi:10.18632/aging.103508)
Supplement: Supplementary Material [file aging-12-103508-s001..pdf]

SUPPLEMENTARY MATERIALS

GLCM

The grey level co-occurrence matrix (GLCM) [Haralick] takes into account the ar-rangements of pairs of voxels to calculate textural indices. The GLCM is calculated

from 13 different directions in 3D with a δ-voxel distance ($\|\underset{d}{\rightarrow}\|$) relationship between neighboured voxels. The index value is the average of the index over the 13 directions in space (X, Y, Z). Seven textural indices are computed from this matrix. An entry (i, j) of GLCM for one direction is equal to:

$$GLCM_{\Delta x,\Delta y}(i, j) = \frac{1}{Pairs\,ROI} \sum_{p=1}^{N-\Delta x} \sum_{q=1}^{M-\Delta y} \begin{cases} 1 if\ \ (I(p,q)=i,\ I\left(p+\Delta x, q+\Delta y\right)=j) \\ \ and\ I(p,q), I\left(p+\Delta x, q+\Delta y\right) \in ROI \\ 0\, otherwise \end{cases}$$

where I (p, q) corresponds to voxel (p, q) in an image (I) of size N*M. The vector $\underset{d}{\rightarrow}$ = (Δx, Δy) covers the 4 directions (D1, D2, D3, D4, Figure 1.1) in 2D space or 13 directions (D1, D2, ..., D13, Figure 1.2) in 3D space and Pairs ROI corresponds to the number of all voxel pairs belonging to the region of interest (ROI).

GLCM_Homogeneity

is the homogeneity of grey-level voxel pairs.

GLCM_Homogeneity = Average over 13 (or 4) directions $\left(\sum_i \sum_j \frac{GLCM(i,j)}{1+|i-j|}\right)$

GLCM_Energy

also called Uniformity or Second Angular Moment, is the uniformity of grey-level voxel pairs.

GLCM_Energy = Average over 13 (or 4) directions $\left(\sum_i \sum_j GLCM(i,j)^2\right)$

GLCM_Contrast

also called Variance or Inertia, is the local variations in the GLCM.

GLCM_Contrast = Average over 13 (or 4) directions $\left(\sum_i \sum_j (i-j)^2 GLCM(i,j)\right)$

GLCM_Correlation

is the linear dependency of grey-levels in GLCM.

GLCM_Correlation = Average over 13 (or 4) directions $\left(\sum_i \sum_j \frac{(i-\mu i)\cdot(j-\mu j)\cdot GLCM(i,j)}{\sigma i \cdot \sigma j}\right)$

where μi or μj corresponds to the average on row i or column j and σi and σj correspond to the variance on row i or column j.

GLCM_Entropy_log10

is the randomness of grey-level voxel pairs.

GLCM_Entropylog10 = Average over 13 (or 4) directions $\left(-\sum_i \sum_j GLCM(i,j) \cdot \log 2(GLCM(i,j)) + \in\right)$

where ε = 2e-16.

Be aware of the logarithm used in the formula.

GLCM_Entropy_log2

is the randomness of grey-level voxel pairs.

GLCM_Entropylog2 = Average over 13 (or 4) direction $\left(-\sum_i \sum_j GLCM(i,j) \cdot \log 10(GLCM(i,j)) + \in\right)$

where ε = 2e-16.

GLCM_Dissimilarity

is the variation of grey-level voxel pairs.

GLCM_Dissimilarity = Average over 13 (or 4) directions $\left(\sum_i \sum_j |i-j| \cdot GLCM(i,j)\right)$

NGLDM

The neighborhood grey-level different matrix (NGLDM) [Amadasum1989] corresponds to the

difference of grey-levels between one voxel and its 26 neighbours in 3 dimensions (8 in 2D). Three texture indices can be computed from this matrix. An element (i, 1) of NGLDM corresponds to the probability of occurrence of level i and an element (i, 2) is equal to:

$$\text{NGLDM}(i,2) = \sum_p \sum_q \begin{cases} |\bar{M}(p,q) - i| & \text{if } I(p,q) = i \\ 0 & \text{else} \end{cases}$$

where $\bar{M}$(p, q) is the average of intensities over the 26 neighbor voxels of voxel (p, q).

NGLDM_Coarseness

is the level of spatial rate of change in intensity.

$$\text{NGLDM\_Coarseness} = \frac{1}{\sum_i NGLDM(i,1) \cdot NGLDM(i,2)}$$

NGLDM_Contrast

is the intensity difference between neighbouring regions.

$$\text{NGLDM\_Contrast} = \left[\sum_i \sum_j NGLDM(i,1) \cdot NGLDM(j,1) \cdot (i, j)^2\right] \cdot \frac{\sum_i NGLDM(i,2)}{E \cdot G \cdot (G-1)}$$

where E corresponds to the number of voxels in the Volume of Interest and G the number of grey-levels.

NGLDM_Busyness

is the spatial frequency of changes in intensity.

$$\text{NGLDM\_Busyness} = \frac{\sum_i NGLDM(i,1) \cdot NGLDM(i,2)}{\sum_i \sum_j |(i \cdot NGLDM(i,1) - j \cdot NGLDM(j,1) \cdot (i, j)^2)|}$$

with NGLDM (i, 1) ≠ 0, NGLDM(j, 1) ≠ 0

GLRLM

The grey-level run length matrix (GLRLM) [Xu] gives the size of homogeneous runs for each grey level. This matrix is computed for the 13 different directions in 3D (4 in 2D) and for each of the 11 texture indices derived from this matrix, the 3D value is the average over the 13 directions in 3D (4 in 2D). The element (i, j) of GLRLM corresponds to the number of homogeneous runs of j voxels with intensity i in an image and is called GLRLM(i, j) thereafter.

GLRLM_SRE, GLRLM_LRE

Short-Run Emphasis or Long-Run Emphasis is the distribution of the short or the long homogeneous runs in an image.

GLRLM_SRE = Average over 13 (or 4) directions

$$\left(\frac{1}{H} \sum_i \sum_j \frac{GLRLM(i, j)}{i^2}\right)$$

GLRLM_LRE = Average over 13 (or 4) directions

$$\left(\frac{1}{H} \sum_i \sum_j GLRLM(i, j) \cdot i^2\right)$$

GLRLM_SRLGE, GLRLM_SRHGE

Short-Run Low Gray-level Emphasis or Short-Run High Gray-level Emphasis is the distribution of the short homogeneous runs with low or high grey-levels.

GLRLM_SRLGE = Average over 13 (or 4) directions

$$\left(\frac{1}{H} \sum_i \sum_j \frac{GLRLM(i, j)}{i^2 \cdot j^2}\right)$$

GLRLM_SRHGE = Average over 13 (or 4) directions

$$\left(\frac{1}{H} \sum_i \sum_j \frac{GLRLM(i, j) \cdot i^2}{j^2}\right)$$

GLRLM_LRLGE, GLRLM_LRHGE

Long-Run Low Gray-level Emphasis or Long-Run High Gray-level Emphasis is the distribution of the long homogeneous runs with low or high grey-levels.

GLRLM_LRLGE = Average over 13 (or 4) directions

$$\left(\frac{1}{H} \sum_i \sum_j \frac{GLRLM(i, j) \cdot j^2}{i^2}\right)$$

GLRLM_LRHGE = Average over 13 (or 4) directions

$$\left(\frac{1}{H} \sum_i \sum_j GLRLM(i, j) \cdot i^2 \cdot j^2\right)$$

GLRLM_GLNUr, GLRLM_RLNU

Gray-Level Non-Uniformity for run or Run Length Non-Uniformity is the nonuniformity of the grey-levels or the length of the homogeneous runs.

GLRLM_GLNUr = Average over 13 (or 4) directions

$$\left(\frac{1}{H} \sum_i \left(\sum_j GLRLM(i, j)\right)^2\right)$$

GLRLM_RLNU = Average over 13 (or 4) directions $\left(\frac{1}{H}\sum_j\left(\sum_i GLRLM(i,j)\right)^2\right)$

GLRLM_RP

Run Percentage measures the homogeneity of the homogeneous runs.
GLRLM_RP = Average over 13 (or 4) directions $\left(\frac{H}{\sum_i\sum_j\left(j\cdot GLRLM(i,j)\right)}\right)$

GLZLM

The grey-level zone length matrix (GLZLM) Thibault] provides information on the size of homogeneous zones for each grey-level in 3 dimensions (or 2D). It is also named Grey Level Size Zone Matrix (GLSZM). From this matrix, 11 texture indices are computed. Element (i, j) of GLZLM corresponds to the number of homogeneous zones of j voxels with the intensity i in an image and is called GLZLM(i, j) thereafter.

GLZLM_SZE, GLZLM_LZE

Short-Zone Emphasis or Long-Zone Emphasis is the distribution of the short or the long homogeneous zones in an image.

$$\text{GLZLM\_SZE} = \frac{1}{H}\sum_i\sum_j\frac{GLZLM(i,j)}{j^2}$$

$$\text{GLZLM\_LZE} = \frac{1}{H}\sum_i\sum_j GLZLM(i,j)\cdot j^2$$

where H corresponds to the number of homogeneous zones in the Volume of Interest.

GLZLM_LGZE, GLZLM_HGZE

Low Gray-level Zone Emphasis or High Gray-level Zone Emphasis is the distribution of the low or high grey-level zones.

$$\text{GLZLM\_LGZE} = \frac{1}{H}\sum_i\sum_j\frac{GLZLM(i,j)}{i^2}$$

$$\text{GLZLM\_HGZE} = \frac{1}{H}\sum_i\sum_j GLZLM(i,j)\cdot i^2$$

GLZLM_SZLGE, GLZLM_SZHGE

Short-Zone Low Gray-level Emphasis or Short-Zone High Gray-level Emphasis is the distribution of the short homogeneous zones with low or high grey-levels.

$$\text{GLZLM\_SZLGE} = \frac{1}{H}\sum_i\sum_j\frac{GLZLM(i,j)}{i^2\cdot j^2}$$

$$\text{GLZLM\_SZHGE} = \frac{1}{H}\sum_i\sum_j\frac{GLZLM(i,j)\cdot i^2}{j^2}$$

GLZLM_LZLGE, GLZLM_LZHGE

Long-Zone Low Gray-level Emphasis or Long-Zone High Gray-level Emphasis is the distribution of the long homogeneous zones with low or high grey-levels.

$$\text{GLZLM\_LZLGE} = \frac{1}{H}\sum_i\sum_j\frac{GLZLM(i,j)\cdot j^2}{i^2}$$

$$\text{GLZLM\_LZHGE} = \frac{1}{H}\sum_i\sum_j GLZLM(i,j)\cdot i^2\cdot j^2$$

GLZLM_GLNUz, GLZLM_ZLNU

Gray-Level Non-Uniformity for zone or Zone Length Non-Uniformity is the nonuniformity of the grey-levels or the length of the homogeneous zones.

$$\text{GLZLM\_GLNUz} = \frac{1}{H}\sum_i\left(\sum_j GLZLM(i,j)\right)^2$$

$$\text{GLZLM\_ZLNU} = \frac{1}{H}\sum_j\left(\sum_i GLZLM(i,j)\right)^2$$

GLZLM_ZP

Zone Percentage measures the homogeneity of the homogeneous zones.

$$\text{GLZLM\_ZP} = \frac{H}{\sum_i\sum_j\left(j\cdot GLZLM(i,j)\right)}$$

SHAPE_SPHERICITY

is how spherical a Volume of Interest is. Sphericity is equal to 1 for a perfect sphere.

$$\text{SHAPE\_Sphericity} = \frac{\pi^{1/3\cdot(6V)^{2/3}}}{A}$$

where V and A correspond to the volume and the surface of the Volume of Interest based on the Delaunay triangulation.

SHAPE_Compacity

reflects how compact the Volume of Interest is.

$$\text{SHAPE\_Compacity} = \frac{A^{3/2}}{V}$$

where V and A correspond to the volume and the surface of the Volume of Interest based on the Delaunay triangulation.

SHAPE_Volume (mL and voxels)

is the Volume of Interest in mL and in voxels.

$$\text{SHAPE\_Volume} = \sum_i V_i$$

where Vi corresponds to the volume of voxel i of the Volume of Interest.

HISTOGRAM CALCULATION

To build a histogram HISTO, it is necessary to determine a bin width ("bin" parameter). The indices derived from the histogram will depend on this bin width parameter.

HISTO_Skewness

is the asymmetry of the grey-level distribution in the histogram.

$$\text{HISTO\_Skewness} = \frac{\frac{1}{E}\sum_i \left(HISTO(i) - \overline{HISTO}\right)^3}{\left(\sqrt{\frac{1}{E}\sum_i \left(HISTO(i) - \overline{HISTO}\right)^2}\right)^3}$$

where HISTO(i) corresponds to the number of voxels with intensity i, E the total number of voxels in the Volume of Interest and $\overline{\text{HISTO}}$ the average of grey-levels in the histogram.

HISTO_Kurtosis

reflects the shape of the grey-level distribution (peaked or flat) relative to a normal distribution.

$$\text{HISTO\_Kurtosis} = \frac{\frac{1}{E}\sum_i \left(HISTO(i) - \overline{HISTO}\right)^4}{\left(\frac{1}{E}\sum_i \left(HISTO(i) - \overline{HISTO}\right)^2\right)^2}$$

where HISTO(i) corresponds to the number of voxels with intensity i, E the total number of voxels in the Volume of Interest and $\overline{HISTO}$ the average of grey-levels in the histogram

HISTO_Entropy_log10

reflects the randomness of the distribution.

$$\text{HISTO\_Entropylog10} = -\sum_i p(i) \cdot log_{10}\left(p(i) + \varepsilon\right)$$

where p(i) is the probability of occurrence of voxels with intensity i and ε = 2e-16.

HISTO_Entropy_log2

reflects the randomness of the distribution.

$$\text{HISTO\_Entropylog2} = -\sum_i p(i) \cdot log_2\left(p(i) + \varepsilon\right)$$

where p(i) is the probability of occurrence of voxels with intensity i and ε = 2e-16.

HISTO_Energy

reflects the uniformity of the distribution.

$$\text{HISTO\_Energy} = \sum_i p(i)^2$$
