# Supplementary material for: Radiomics-based prediction of survival in patients with head and neck squamous cell carcinoma based on pre- and post-treatment 18F-PET/CT
Source: Aging (Albany NY). 2020 Jul 16;12(14):14593–619. doi: 10.18632/aging.103508 (PMC7425452; doi:10.18632/aging.103508)
Supplement: Supplementary Figures [file aging-12-103508-s003..pdf]

SUPPLEMENTARY FIGURES

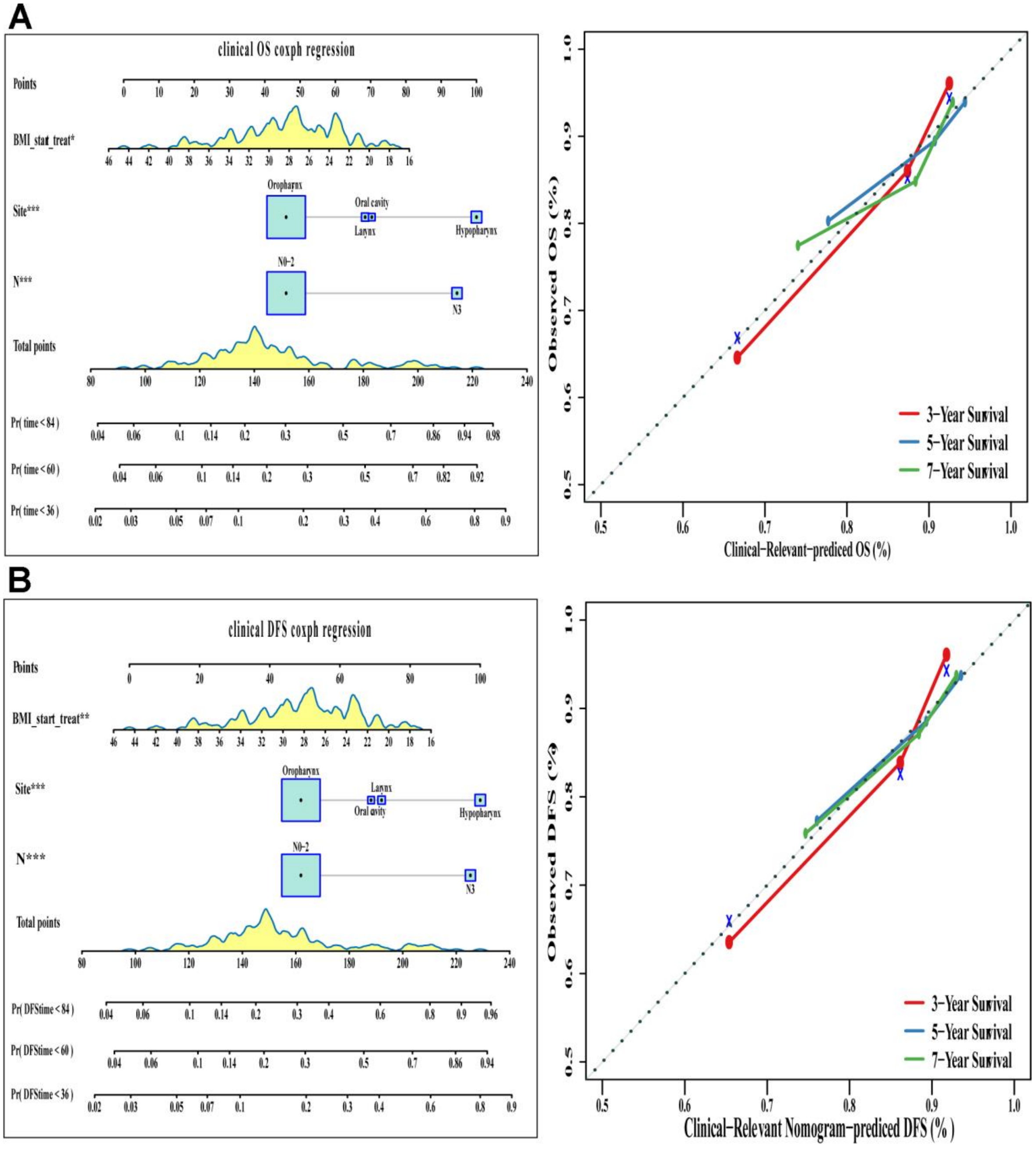

Supplementary Figure 1. The clinical-relevant survival nomograms and their calibration plots. (**A**) OS-based model. (**B**) DFS-based model.

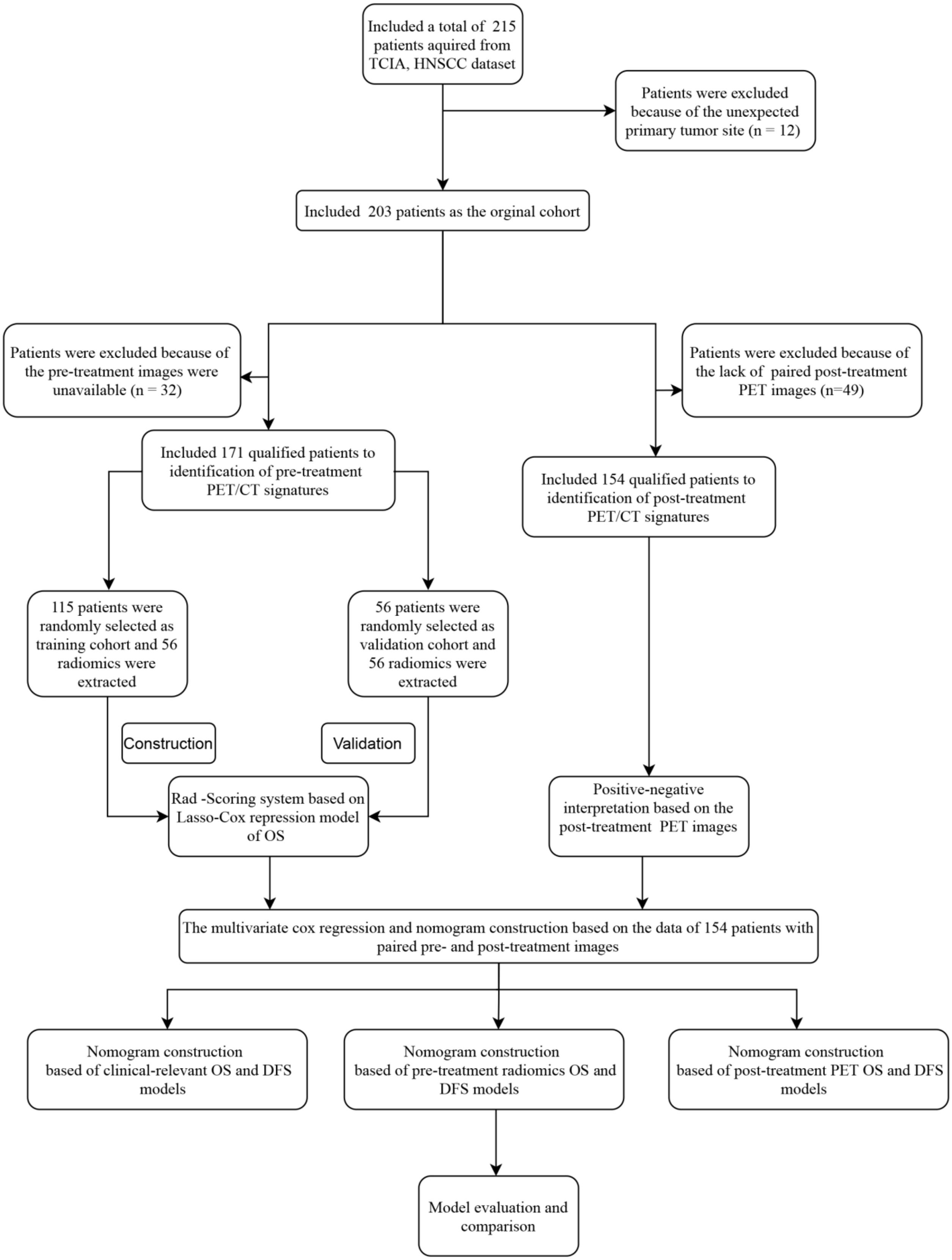

Supplementary Figure 2. The flow chart of the study design.
