# Supplementary material for: Radiomics-based prediction of survival in patients with head and neck squamous cell carcinoma based on pre- and post-treatment 18F-PET/CT
Source: Aging (Albany NY). 2020 Jul 16;12(14):14593–619. doi: 10.18632/aging.103508 (PMC7425452; doi:10.18632/aging.103508)
Supplement: Supplementary Tables [file aging-12-103508-s002..pdf]

SUPPLEMENTARY TABLES

Supplementary Table 1. Univariate and multivariate Cox regression analyses for OS and DFS in the radiomics features.

Variables	Overall survival		Disease-free survival	
	HR (95%CI)	*p*	HR (95%CI)	*p*
Univariate analysis				
SHAPE_Sphericity	0.69 (0.48-1.01)	0.056	0.70 (0.49-1.02)	0.064
NGLDM_Coarseness	1.24 (0.96-1.61)	0.100	1.22 (0.95-1.58)	0.119
SMTV	1.42 (0.99-2.03)	0.060	1.48 (1.01 - 2.16)	0.046
Multivariate analysis				
SHAPE_Sphericity	0.71 (0.49-1.04)	0.081	0.72 (0.50-1.06)	0.096
NGLDM_Coarseness	1.45 (1.11-1.90)	0.003	1.43 (1.10-1.87)	0.008
SMTV	1.57 (1.05-2.33)	0.027	1.64 (1.08-0.49)	0.021

Abbreviations: OS-overall survival; DFS-disease-free survival; HR-hazard ratio; NGLDM-neighborhood grey-level different matrix; SMTV-standardized metabolic tumor value;

Supplementary Table 2. Clinical characteristics of patients according to the Rad-score in the combined training and validation cohorts.

Variables	Combined cohort (N = 171)			*p*-value
	N	Low Radscore	High Radscore	*p*
Gender				0.789
Male	147	96 (65.3%)	51 (34.7%)	
Female	24	15 (62.5%)	9 (37.5%)	
Age (years)				0.036
< 60	101	72 (71.3%)	29 (28.7%)	
≥ 60	70	39 (55.7%)	31 (44.3%)	
Tumor size				0.005
≤ 4	85	64 (75.3%)	21 (24.7%)	
> 4	86	47 (54.7)	39 (45.3%)	
Tumor Location				
Oropharynx	137	91 (66.4%)	46 (33.6%)	0.538
Larynx	19	12 (63.2%)	7 (36.8%)	
Oralcavity	4	3 (75.0%)	1 (25.0%)	
Hypopharynx	11	5 (45.5%)	6 (54.5%)	
Differentiation status				0.234
Well	17	4 (23.5%)	13 (76.5%)	
Moderate	79	54 (68.4%)	25 (31.6%)	
Poor and undifferentiated	75	44 (58.7%)	31 (41.3%)	
T Stage*				< 0.001
T1	31	20 (64.5%)	11 (35.5%)	
T2	54	44 (81.5%)	10 (18.5%)	
T3	52	36 (69.2%)	16 (30.8%)	
T4	34	11 (32.4%)	23 (67.6%)	
N Stage*				
N0	19	14 (73.7%)	5 (26.3%)	0.066
N1	19	14 (73.7%)	5 (26.3%)	
N2a	9	9 (100%)	0 (0%)	
N2b	75	46 (61.3%)	29 (38.7%)	
N2c	37	24 (64.9%)	13 (35.1%)	
N3	12	4 (33.3)	8 (66.7%)	
TNM stage*				0.044
I	1	1 (100%)	0 (0%)	
II	5	5 (100%)	0 (0%)	
III	28	22 (78.6%)	6 (21.4%)	
IVA	122	77 (63.1%)	45 (36.9%)	
IVB	15	6 (40%)	9 (60%)	

* according to 7th AJCC stage system.

Supplementary Table 3. Univariate Cox regression analyses for OS and DFS in the combined cohort.

Variables	Overall survival		Disease-free survival	
	**HR (95%CI)**	***p***	**HR (95%CI)**	***p***
**Univariate Cox Regression Analysis**				
**PET outcome (vs. negative)**	6.60 (3.65-11.97)	<0.001	8.17 (4.45-15.00)	<0.001
**Rad-score**	6.43 (2.68-15.4)	<0.001	6.81 (2.75-16.85)	<0.001
**Start-treatment BMI**	0.90 (0.85-0.97)	0.002	0.90 (0.85-0.96)	<0.001
**Cancer site (vs. Oropharynx)**				
Hypopharynx	5.85 (2.55-13.44)	<0.001	5.43 (2.37-12.39)	<0.001
Oral cavity	1.69 (0.23-12.54)	0.606	1.57 (0.21-11.6)	0.658
Larynx	2.68 (0.80-9.70)	0.114	2.58 (0.75-8.90)	0.135
**N stage (vs. N0-2)**	3.08 (1.36-6.97)	0.007	2.95 (1.29-6.73)	0.010
**Age**	1.05 (1.02-1.09)	0.001	1.05 (1.02-1.08)	0.002
**Histologic grade (vs. well-moderate differentiated)**	0.57 (0.31-1.06)	0.076	0.56 (0.30-1.05)	0.071
**T stage (vs. T1-3)**	1.76 (0.89-3.49)	0.105	1.66 (0.84-3.29)	0.146
**7th AJCC Stage (vs. stage I-III)**	0.68 (0.35-1.35)	0.273	0.66 (0.33-1.31)	0.232
**Smoking history (vs. never)**				
Less than 10 pack-years	1.43 (0.52-3.92)	0.485	1.51 (0.55-4.11)	0.422
greater or equal than 10 pack-years	1.13 (0.60-2.13)	0.705	1.13 (0.60-2.13)	0.699
**Induction Chemotherapy (vs. no)**	0.93 (0.44-1.55)	0.557	0.82 (0.44-1.53)	0.526
**Concurrent Chemoradiotherapy (vs. no)**	1.07 (0.59-1.95)	0.818	1.07 (0.59-1.94)	0.829

Abbreviations: OS-overall survival; DFS-disease free survival; HR-hazard ratio; BMI-body mass index; PET- positron emission tomography/computed tomography.

Supplementary Table 4. Univariate and multivariate Cox regression analyses for OS and DFS in the radiomics model and traditional PET model.

Variables	Overall survival			Disease-free survival		
	**HR (95%CI)**	***p***	**C-index**	**HR (95%CI)**	***p***	**C-index**
Rad-score	6.332 (2.765-14.5)	< 0.001	**0.641**	6.844 (2.904-16.13)	< 0.001	**0.643**
**Conventional PET features**						
Multivariate analysis			**0.588**			**0.576**
MTV	1.4069 (0.8850-2.236)	0.149		1.335 (0.826-2.159)	0.238	
TLG	0.9903 (0.558-1.758)	0.973		1.061 (0.592-1.902)	0.844	
SUV_{max}	0.7968 (0.442-1.437)	0.450		0.855 (0.477-1.534)	0.600	
SUV_{mean}	1.2836 (0.612-2.694)	0.509		1.141 (0.541-2.407)	0.729	
Univariate analysis						
MTV	1.408 (1.123-1.764)	0.003	0.567	1.400 (1.109-1.768)	0.004	0.562
TLG	1.286 (1.055-1.567)	0.013	0.579	1.281 (1.049-1.565)	0.015	0.580
SUV_{max}	1.045 (0.8141-1.341)	0.731	0.501	1.034 (0.805-1.329)	0.792	0.511
SUV_{mean}	1.105 (0.863-1.415)	0.430	0.530	1.082 (0.844-1.387)	0.535	0.536

Abbreviations: OS-overall survival; DFS-disease-free survival; HR-hazard ratio; PET- positron emission tomography/computed tomography; C-index-Concordance index; MTV-metabolic tumor value; TLG-total lesion glycolysis; SUV- standardized uptake values.

Supplementary Table 5. The image features extracted from PET/CT images of HNSCC patients.

PET paremeters	Intensity features	Shape	GLCM	GLRLM	NGTDM	GLZLM
SUV_{min}	HISTO_Skewness	SHAPE_Volume_ml	GLCM_Homogeneity	GLRLM_SRE	NGLDM_Coarseness	GLZLM_SZE
SUV_{mean}	HISTO_Kurtosis	SHAPE_Volume_vx	GLCM_Energy	GLRLM_LRE	NGLDM_Contrast	GLZLM_LZE
SUV_{std}	HISTO_ExcessKurtosis	SHAPE_Sphericity	GLCM_Contrast	GLRLM_LGRE	NGLDM_Busyness	GLZLM_LGZE
SUV_{max}	HISTO_Entropy_log10	SHAPE_Compacity	GLCM_Correlation	GLRLM_HGRE		GLZLM_HGZE
SUV_{sum}	HISTO_Entropy_log2		GLCM_Entropy_log10	GLRLM_SRLGE		GLZLM_SZLGE
SUVQ1	HIOTO_Energy		GLCM_Entropy_log2	GLRLM_SRHGE		GLZLM_SZHGE
SUVQ2			GLCM_Dissimilarity	GLRLM_LRLGE		GLZLM_LZLGE
SUVQ3				GLRLM_LRHGE		GLZLM_LZHGE
SUVpeak.sphere.0.5mL				GLRLM_GLNU		GLZLM_GLNU
SUVpeak.sphere.1mL				GLRLM_RLNU		GLZLM_ZLNU
MTV				GLRLM_RP		GLZLM_ZP
SMTV						
TLG						
STLG						
